# Risk factors for distant metastasis and prognosis in stage T1 esophageal cancer: A population-based study

**DOI:** 10.3389/fsurg.2022.988460

**Published:** 2023-01-06

**Authors:** Kai Zhu, Mingyue Jia, Linlin Ji, Guangshun Wang

**Affiliations:** ^1^ Department of Thoracic Surgery, Tianjin Baodi Hospital, Baodi Clinical College of Tianjin Medical University, Tianjin, China; ^2^ Department of Obstetrics and Gynecology, Jinan Central Hospital Affiliated to Shandong First Medical University, Jinan, China

**Keywords:** esophageal cancer, stage T1, SEER database, nomogram, distant metastasis

## Abstract

**Purpose:**

Stage T1 esophageal cancer (EC) with distant metastasis (DM) is rare and poorly understood. In this study, we aimed to construct and validate a novel nomogram for predicting the probability of DM in T1 EC patients.

**Methods:**

A total of 1,663 eligible T1 EC patients were enrolled from the Surveillance, Epidemiology, and End Results (SEER) database between 2004 and 2015. The patients were randomly divided into training and validation cohorts. Univariate and multivariate logistic analyses in the training cohort were used to identify risk factors related to DM, and then these risk factors were applied to construct the nomogram. Receiver operating characteristic (ROC) curves, the area under the curve (AUC), calibration plots, the Hosmer-Lemeshow (HL) test, and decision curve analysis (DCA) were used to evaluate the nomogram.

**Results:**

Among the 1,663 patients identified, 143 (8.6%) had DM. Five risk factors (tumor location, lymph node status, tumor length, T1 subtype, and grade) were significant predictors of DM. The AUC values were 0.828 and 0.851 in the training cohort and validation cohort, respectively, revealing good discrimination. The calibration plots in the training cohort and validation cohort both showed good consistency. DCA showed that the nomogram was clinically effective. In addition, the nomogram has a good risk stratification ability to identify patients with different risks according to the nomogram score. In terms of survival analysis, univariate and multivariate Cox analyses showed that age, race, tumor length, grade, lymph node status, M stage and treatment were significant prognostic factors for overall survival (OS). For cancer-specific survival (CSS), the independent prognostic factors were age, tumor length, histology, grade, lymph node status, M stage and treatment.

**Conclusion:**

The nomogram could effectively predict the probability of DM in T1 EC patients. It can aid clinicians in detecting high-risk patients and making individual clinical decisions.

## Introduction

According to the latest global cancer statistics, esophageal cancer (EC) is still one of the most common malignant tumors. Approximately 604,000 new esophageal cancer cases and 544,000 deaths occurred in 2020, and its morbidity and mortality rank seventh and sixth, respectively (1). The treatment strategy of EC mainly depends on its American Joint Commission on Cancer (AJCC) staging. For T1 EC, most patients can receive surgery or endoscopic treatment and usually have a good prognosis (2). However, if distant metastasis (DM) occurs, the situation is not optimistic, although this is very rare in the clinic. Once DM develops, even if the depth of tumor invasion is superficial, surgery is usually not recommended. Generally, only palliative management, such as concurrent chemoradiation or immunotherapy, can be used, and survival is far worse than that without DM (3, 4). Therefore, it is very important to determine whether there is DM when the patient is first diagnosed. For patients with T1 EC, the treatment strategies with and without DM are completely different. If people with a high risk of DM can be identified and further detailed inspections such as PET-CT can be performed, unnecessary operations can be avoided.

Nomograms are visualization tools based on regression models and are currently widely used. They can be used to numerically calculate the risk probability of clinical events for individual patients (5). Previous studies have constructed nomograms to predict the lymph node metastasis rate of T1 EC, which can aid surgical decision making in patients who have undergone endoscopic resection (6, 7). However, no studies have yet focused on DM of T1 EC.

The Surveillance, Epidemiology, and End Results (SEER) database is an authoritative source for cancer statistics in the United States. It collects cancer diagnosis, treatment and survival data for approximately 30% of the U.S. population. Therefore, SEER can be used to analyze cancer data using a large sample size. Because T1 EC with DM is rare in the clinic, it is difficult to collect multicenter data. Therefore, this study aimed to use the SEER database to construct and validate a novel nomogram for predicting DM in T1 EC patients.

## Materials and methods

### Patient enrollment and clinical characteristics

We used SEER*Stat 8.3.9 software to download esophageal cancer patient data from the SEER 18 registries between 2004 and 2015. The exported data included the following variables: year of diagnosis, ICD-O-3 Hist/behav, sex, age, race, grade, tumor location, tumor length, T stage, N stage, M stage, treatment (including surgery, radiotherapy and chemotherapy), survival status, cause of death, and survival months. All patients with stage M1 had at least one organ metastasis at diagnosis.

**Figure 1:**
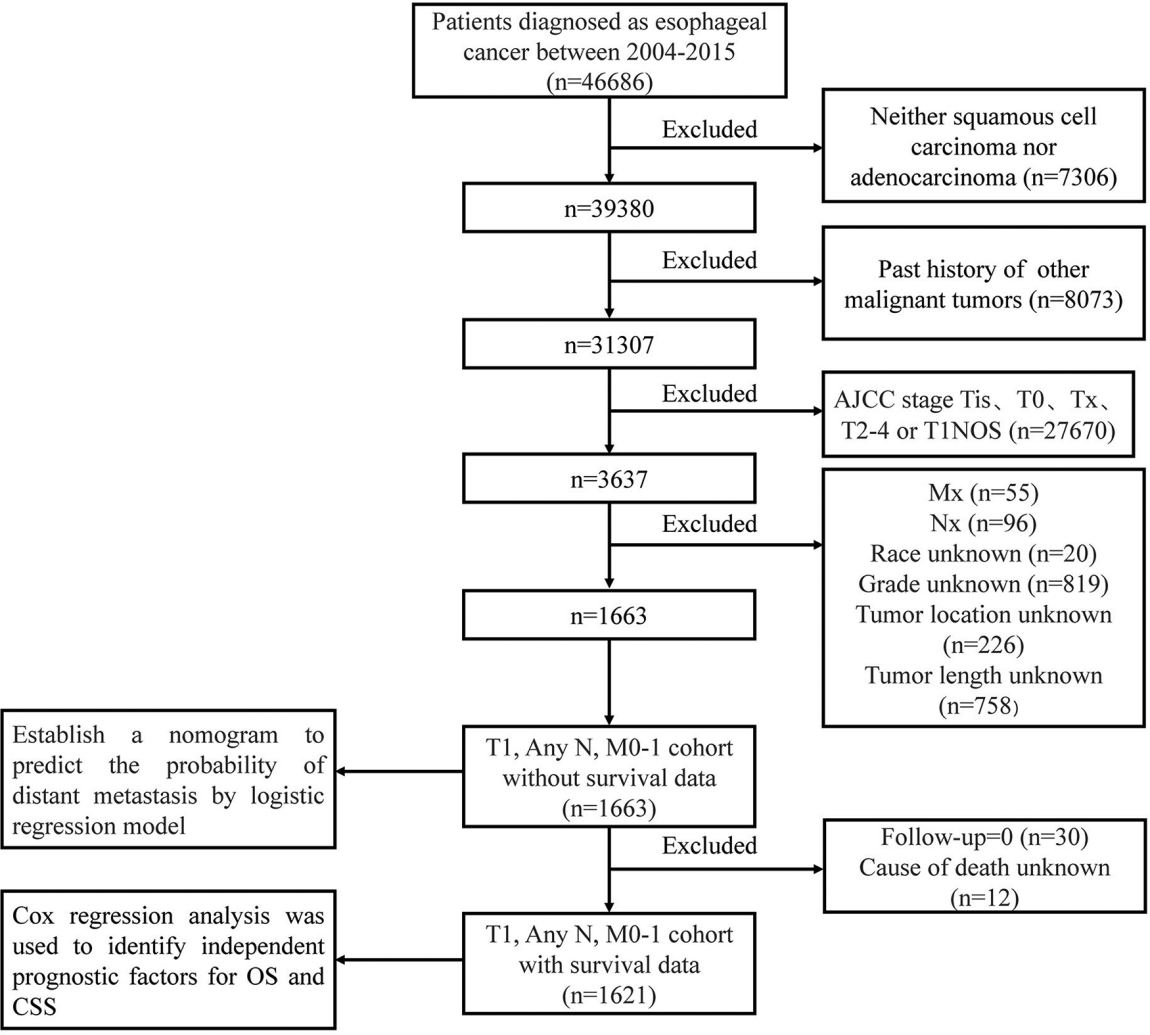
Patient enrollment flowchart.

The inclusion criteria were as follows: (1) pathologically confirmed adenocarcinoma or squamous cell carcinoma, (2) age >18 years old, and (3) AJCC stage T1. The exclusion criteria were as follows: (1) past history of other malignant tumors, (2) T1 subtype cannot be determined, (3) data such as race, grade, tumor length, tumor location, N stage, M stage missing or incomplete, (4) follow-up time is 0, (5) cause of death unknown. The flowchart of patient enrollment is shown in Figure 1. The T1 subtype can be divided into T1a and T1b. T1a is defined as the tumor invading the lamina propria or muscularis mucosae, and T1b is defined as the tumor invading the submucosa without infiltration of the muscularis. The TNM staging data were based on the AJCC 7th edition staging system.

### Construction and validation of the nomogram model

We used the “caret” package of R software to randomly divide all patients into training (70%) and validation (30%) cohorts. Univariable and multivariable analyses were performed to identify the independent risk factors for DM in T1 EC in the training cohort. Based on the multivariable binary logistic regression model, we formulated a nomogram by R software. Then, we used the training cohorts for internal validation by the bootstrapping method and the validation cohorts for external validation. Receiver operating characteristic (ROC) curves and the area under the curve (AUC) value were used to evaluate the discrimination of the model, that is, the ability to correctly distinguish patients with DM and without DM. Calibration curves and the Hosmer–Lemeshow (HL) test (8). were used to evaluate the calibration of the model, that is, the degree of consistency between the predicted DM risk and the actual DM risk. Subsequently, we used decision curve analysis (DCA) to calculate the net benefits at each risk threshold probability to evaluate the clinical effectiveness of the model (9).

### Statistical analysis

Chi squared tests were used to compare categorical variables between different groups. The Kaplan–Meier method was used to perform survival analysis, and significance was determined using the log-rank test. A multivariate Cox regression model was used to investigate independent prognostic factors for T1 EC. Statistical significance was considered at *p *< 0.05, but the HL test was statistically significant at *p *> 0.05. All statistical analyses and plots were performed with SPSS software (version 25.0.) and R software (version 4.0.5).

## Results

### Patient characteristics

After screening, a total of 1,663 patients were finally included in our study. We divided the patients into training and validation cohorts, and the demographics and clinicopathological characteristics assessed in the three cohorts are shown in Table 1. DM occurred in 143 of 1,663 patients (8.6%) in the whole cohort. In general, there were approximately the same number of young patients and old patients (50.3% vs. 49.7%). The vast majority of patients were male (83.3%) and white (88.2%). Adenocarcinoma (78.4%) predominated in pathological types, and most tumors were highly differentiated (G1/G2, 66.0%). Most patients had a tumor in the lower 1/3 (74.3%). In terms of the T1 subtype, the number of patients with T1a and T1b was similar (51.7% vs. 48.3%). In addition, there were far more patients without lymph node metastasis (LNM) than those with LNM (78.4% vs. 21.6%). Because most of the patients were staged T1N0M0, they were mainly treated with surgery alone and surgery plus a conservative treatment such as chemotherapy (CT), radiotherapy (RT) or chemoradiotherapy (CRT). According to the results of the chi-square test, there were no statistically significant differences between the training and validation cohorts except in histology (*p *= 0.038).

**Table 1:** Clinicopathologic characteristics of T1 EC patients.

Variables	Whole cohort (*n* = 1663)	Training cohort (*n* = 1167)	Validation cohort (*n* = 496)	*p*-value
Age (years)				0.301
≤65	836 (50.3)	577 (49.4)	259 (52.2)	
>65	827 (49.7)	590 (50.6)	237 (47.8)	
Sex				0.215
Male	1,386 (83.3)	964 (82.6)	422 (85.1)	
Female	277 (16.7)	203 (17.4)	74 (14.9)	
Race				0.148
White	1,466 (88.2)	1,033 (88.5)	433 (87.3)	
Black	115 (6.9)	84 (7.2)	31 (6.3)	
Others	82 (4.9)	50 (4.3)	32 (6.4)	
Tumor length (cm)				0.247
≤2	896 (53.9)	618 (53.0)	278 (56.0)	
>2	767 (46.1)	549 (47.0)	218 (44.0)	
Histology				0.038
Adenocarcinoma	1,304 (78.4)	931 (79.8)	373 (75.2)	
Squamous	359 (21.6)	236 (20.2)	123 (24.8)	
Grade				0.441
G1/G2	1,097 (66.0)	763 (65.4)	334 (67.3)	
G3/G4	566 (34.0)	404 (34.6)	162 (32.7)	
Tumor location				0.232
Upper 1/3	54 (3.2)	34 (2.9)	20 (4.0)	
Middle 1/3	326 (19.6)	224 (19.2)	102 (20.6)	
Lower 1/3	1,236 (74.3)	871 (74.6)	365 (73.6)	
Overlapping	47 (2.9)	38 (3.3)	9 (1.8)	
T1 subtype				0.534
T1a	859 (51.7)	597 (51.2)	262 (52.8)	
T1b	804 (48.3)	570 (48.8)	234 (47.2)	
Lymph node status				0.758
N0	1,303 (78.4)	912 (78.1)	391 (78.8)		
N^+^	360 (21.6)	255 (21.9)	105 (21.2)	
M status				0.947	
M0	1,520 (91.4)	1,067 (91.4)	453 (91.3)		
M1	143 (8.6)	100 (8.6)	43 (8.7)		
Treatment				0.397	
None or LTD^a^	317 (19.1)	228 (19.5)	89 (17.9)	
S alone	767 (46.1)	535 (45.8)	232 (46.8)	
CT/RT/CRT	348 (20.9)	244 (21.0)	104 (21.0)	
S plus CT/RT/CRT	231 (13.9)	160 (13.7)	71 (14.3)	

M, metastasis; LTD, local tumor destruction; S, surgery; CT, chemotherapy; RT, radiotherapy; CRT, chemoradiotherapy.

^a^ Includes photodynamic therapy, electrocautery, cryosurgery and laser ablation.

### Independent risk factors for DM and construction of the nomogram

We first performed univariate analysis in the training cohort. The results showed that tumor length, histology, grade, tumor location, T1 subtype, and lymph node status were significantly (*p *< 0.05) associated with DM. Then, we incorporated the above factors into the multivariate logistic regression analysis, and the results are listed in Table 2. Patients with longer tumor lengths (≤2 cm vs. >2 cm, OR = 3.915, *p *< 0.001) and poorly differentiated tumors (G1/G2 vs. G3/G4, OR = 1.991, *p *= 0.004) were more likely to have DM. Compared to patients with tumors located in the upper 1/3 of the esophagus, those with tumors located in the lower 1/3 of the esophagus (OR = 4.431, *p *= 0.068) and overlapping lesions (OR = 7.948, *p *= 0.021) had a higher risk of DM. Regarding T1 subtype and lymph node status, T1a and N^+^ were associated with a higher risk of DM (T1a vs. T1b, OR = 0.352, *p *< 0.001; N0 vs. N^+^, OR = 4.677, *p* < 0.001). Given that patients with N^+^ were more prone to developing DM, we then compared the baseline characteristics between patients with T1N0M1 and T1N^+^M1 (Supplementary Table S1). We found that patients with T1N^+^M1 had poor differentiation and more overlapping lesions than those with T1N0M1.

**Table 2:** Logistic regression analysis of the risk factors for DM in the training cohort.

	Univariate analysis		Multivariate analysis	
	OR (95% CI)	*p*-value	OR (95% CI)	*p*-value
Age (years)				
≤65	1			
>65	0.819 (0.543–1.235)	0.341		
Sex				
Male	1			
Female	0.970 (0.562–1.673)	0.913		
Race				
White	1			
Black	1.908 (0.995–3.658)	0.052		
Others	1.272 (0.491–3.291)	0.620		
Tumor length (cm)				
≤2	1		1	
>2	6.297 (3.686–10.758)	<0.001	3.915 (2.210–6.934)	<0.001
Histology				
Adenocarcinoma	1		1	
Squamous	1.606 (1.012–2.549)	0.044	1.670 (0.908–3.071)	0.099
Grade				
G1/G2	1		1	
G3/G4	2.878 (1.896–4.368)	<0.001	1.991 (1.246–3.181)	0.004
Tumor location				
Upper 1/3	1		1	
Middle 1/3	0.906 (0.194–4.235)	0.900	1.512 (0.296–7.720)	0.619
Lower 1/3	1.530 (0.360–6.506)	0.565	4.431 (0.896–21.910)	0.068
Overlapping	5.714 (1.153–28.322)	0.033	7.948 (1.372–46.060)	0.021
T1 subtype				
T1a	1		1	
T1b	0.440 (0.283–0.684)	<0.001	0.352 (0.217–0.571)	<0.001
Lymph node status				
N0	1		1	
N^+^	7.038 (4.574–10.830)	<0.001	4.677 (2.928–7.470)	<0.001

**Figure 2:**
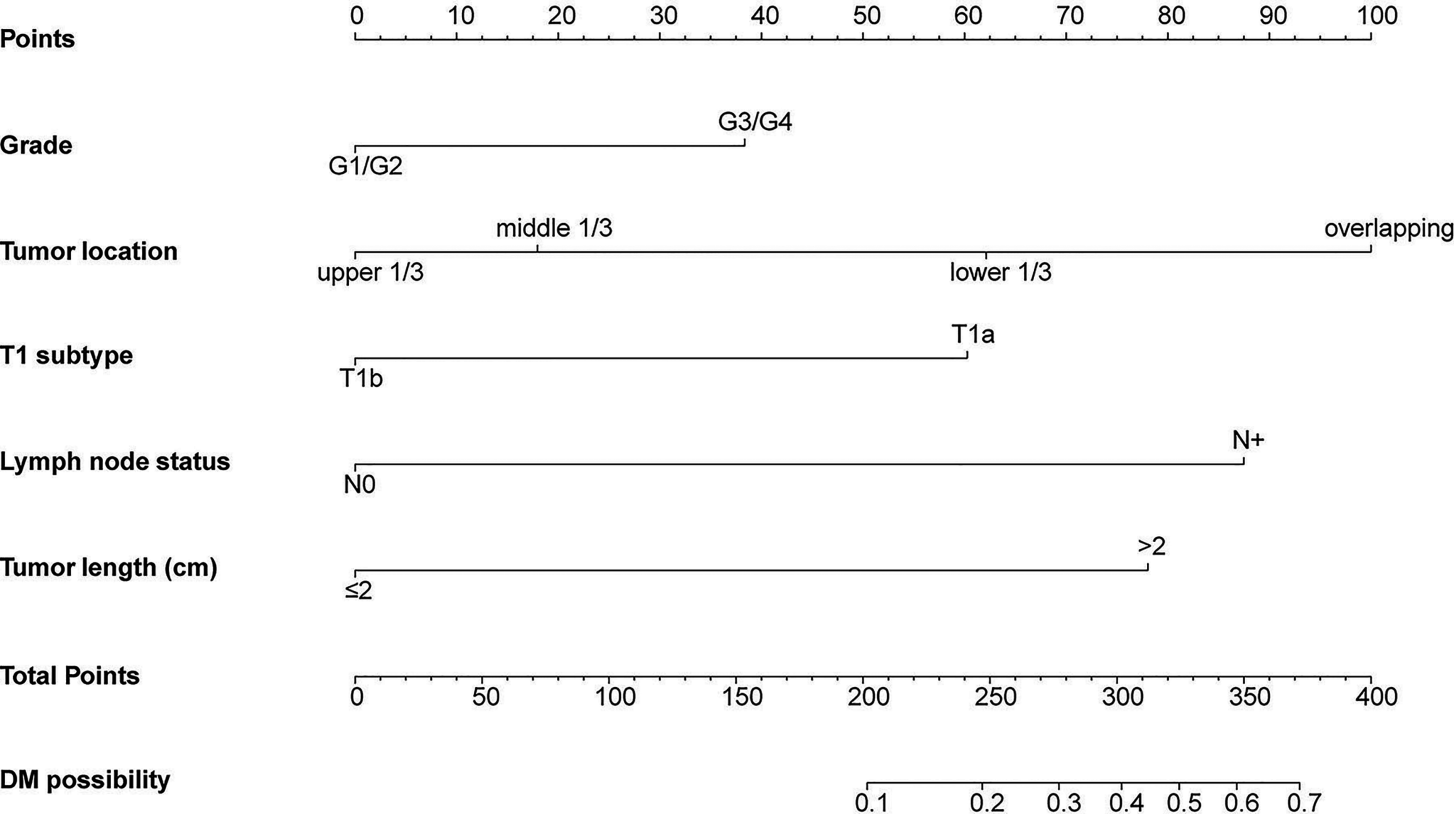
Nomogram for predicting DM in T1 EC patients.

Based on the results of the multivariate logistic regression model, we used five variables to construct a nomogram to predict the probability of DM in T1 EC patients. As shown in Figure 2, tumor location made the largest contribution to the presence of DM according to the length of the line segment, followed by the lymph node status, tumor length, T1 subtype, and grade. The method of using the nomogram is as follows: first, draw a vertical line from each variable to the top points reference line; then, sum the points from each variable to determine a total point on the total points reference line; finally, draw a vertical line from total point to the bottom probability line to obtain the risk of DM of the patients. The nomogram score of each predictive variable is shown in Supplementary Table S2. For example, a patient was assessed under endoscopy ultrasound (EUS) in which the tumor was located in the lower 1/3 of the esophagus with a tumor length of 5 cm, T stage was T1a, and pathological biopsy indicated poor differentiation. In addition, imaging showed the presence of lymph node metastasis. According to the nomogram, the following scores were obtained: 62 for the lower 1/3 of the esophagus, 78 for tumor length >2 cm, 60 for T1a, 38 for poor differentiation, and 87 for N^+^. The total of 325 points corresponds to an approximately 52% chance of DM.

### Validation of the nomogram

**Figure 3:**
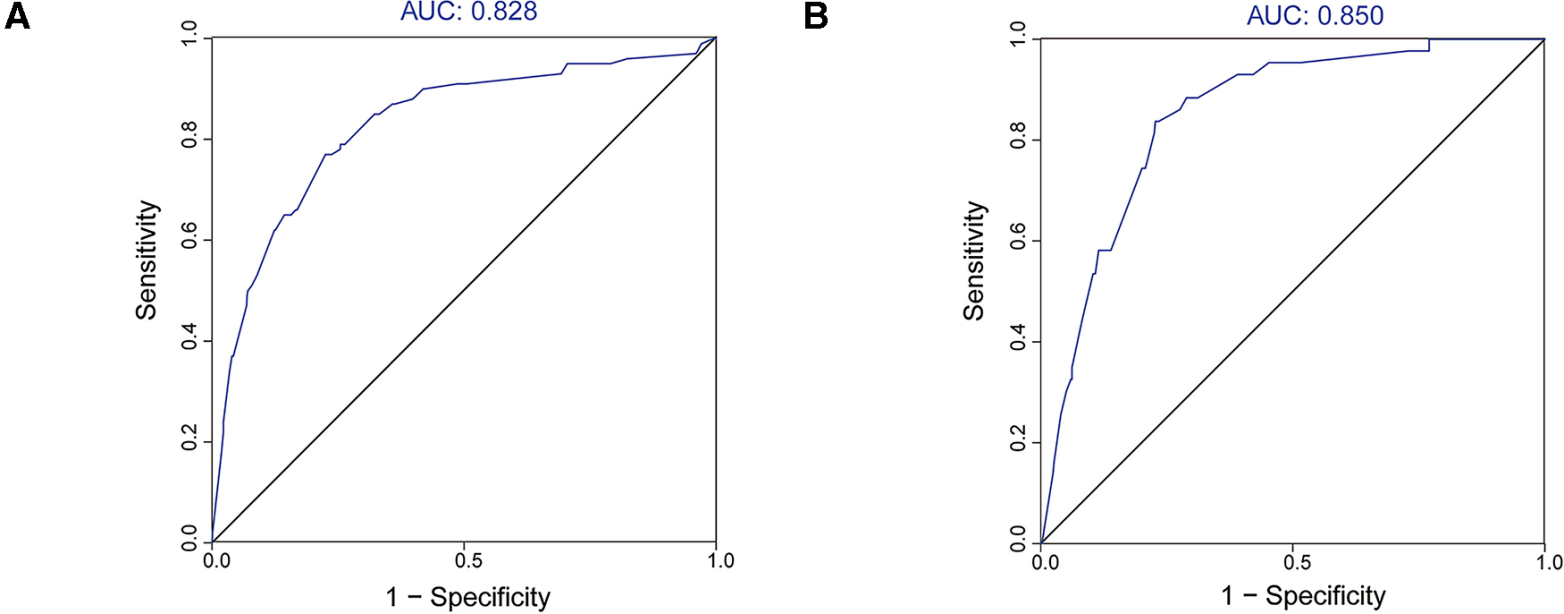
ROC curves and AUC values of the nomogram. (**A**) The training cohort; (**B**) the validation cohort.

We used the ROC curve and its AUC value to evaluate the discrimination of the model (Figure 3). In the training cohort and validation cohort, the AUC values were 0.828 and 0.851, respectively, all of which suggested that the model has a better ability to distinguish patients with DM and without metastases.

**Figure 4:**
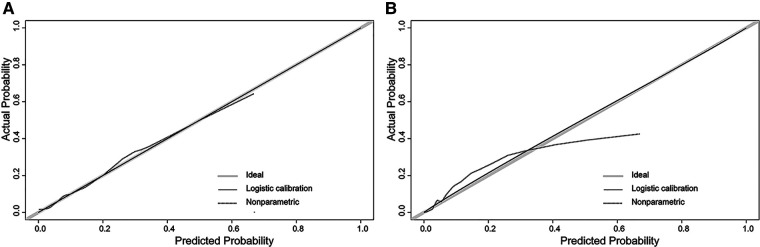
Calibration plot of the nomogram. (**A**) The training cohort; (**B**) the validation cohort. The solid gray line shows that the actual DM probability is equal to the predicted probability. With the solid black line almost coinciding with the solid gray line, the plot reveals excellent calibration in both the training cohort and validation cohort.

**Figure 5:**
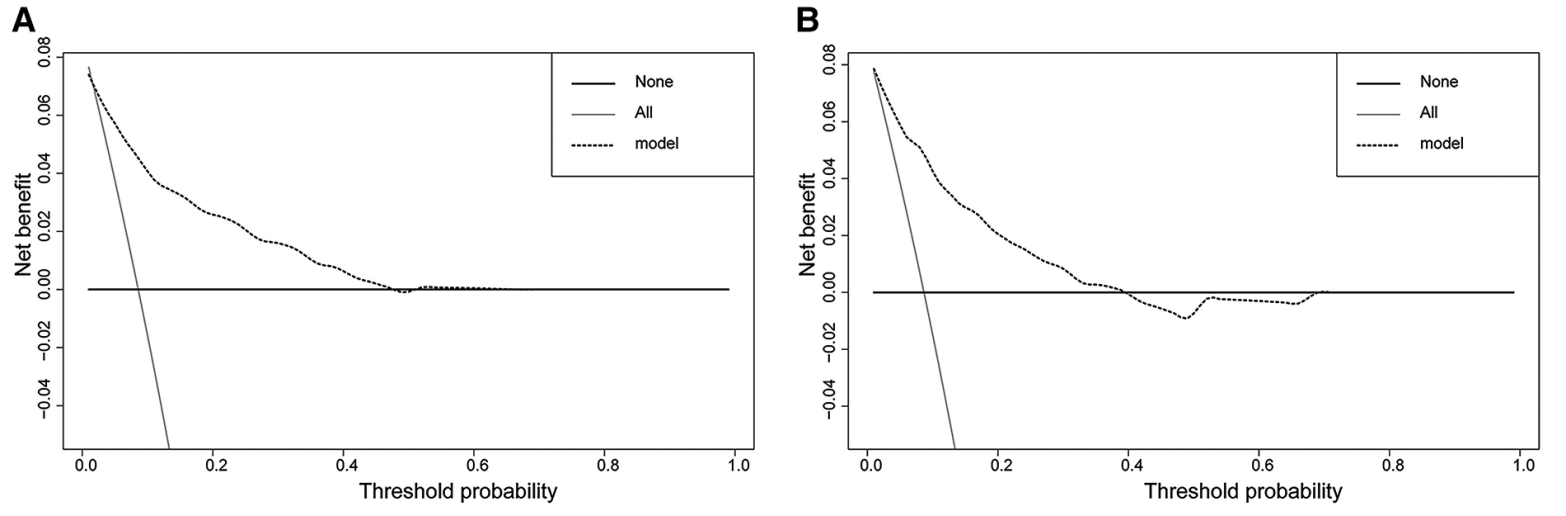
Decision curves of the nomogram. (**A**) The training cohort; (**B**) the validation cohort. The horizontal solid black line represents one extreme situation in which none of the patients experienced DM, and the solid gray line indicates the other extreme situation in which all patients experienced DM. The dotted black line represents the prediction of the nomogram model; the farther away from the above two lines it is, the greater the clinical effectiveness.

In the H-L test, the *p*-value was 0.203 (*p *> 0.05) for the training cohort and 0.345 (*p *> 0.05) for the validation cohort. The calibration plots to predict DM revealed a favorable concordance between the predicted and observed probabilities in the training and validation cohorts, respectively (Figure 4). Furthermore, decision curves showed that threshold probabilities of 0–0.5 and 0–0.4 were the best benefits in the training and validation cohorts (Figure 5). The above results confirmed the effectiveness of the nomogram in predicting DM.

### Survival analysis

We performed survival analysis in the 1,621 patients with survival data. The endpoints were overall survival (OS) and cancer-specific survival (CSS). OS was defined as the time from initial diagnosis to death, regardless of the cause. CSS referred to the period from initial diagnosis to death associated with EC. The median follow-up time was 48 months (range 1–179 months).

**Figure 6:**
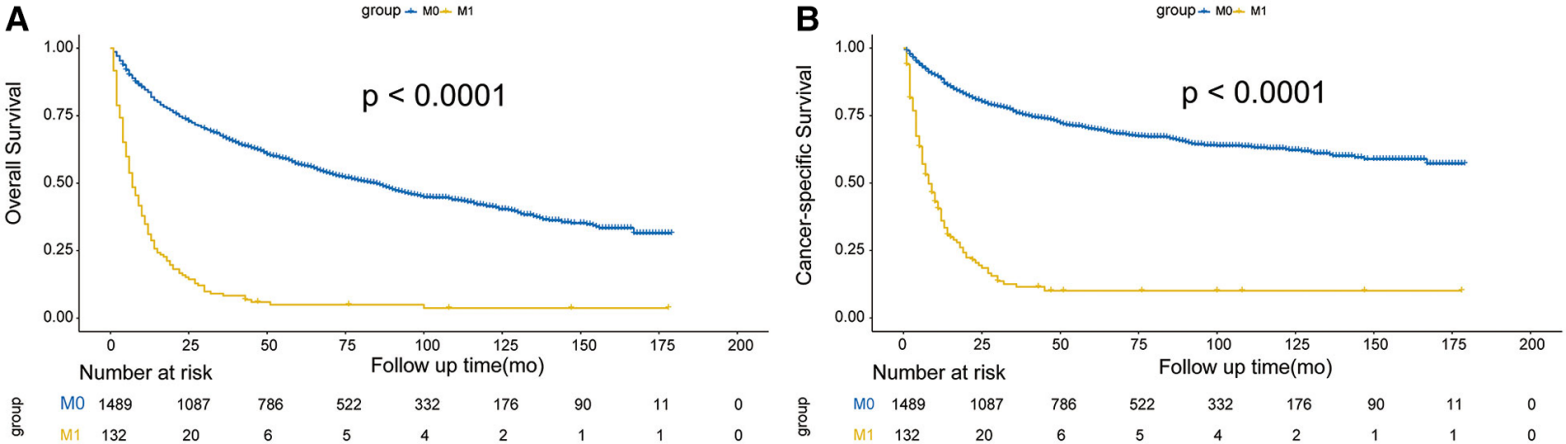
Effect of DM on OS (**A**) and CSS (**B**) in T1 EC.

The one-year, three-year, and five-year OS rates were 84.6%, 67.0%, and 56.9% in M0 patients, respectively, compared with 31.3%, 8.3%, and 5.0% in M1 patients.. Similarly, the one-year, three-year, and five-year CSS rates were 88.5%, 75.9%, and 70.1% in the M1 group, respectively, compared with 40.5%, 11.6%, and 10.1% in the M0 group. Survival curves both showed significant differences in OS (log-rank *χ*^2^ = 363.28, *p *< 0.001) and CSS (log-rank *χ*^2^ = 403.03, *p *< 0.001) (Figure 6).

We used univariate and multivariate Cox regression analyses to screen the independent prognostic factors for OC and CSS. The results are shown in Tables 3, 4. We found that age, race, tumor length, grade, lymph node status, M stage and treatment were significant prognostic factors for OS in T1 EC (Table 3). Similarly, the independent prognostic factors for CSS included age, tumor length, histology, grade, lymph node status, M stage and treatment (Table 4).

**Table 3:** Cox regression analysis of the risk factors for OS in patients with survival data.

	Univariate analysis		Multivariate analysis	
	HR (95% CI)	*p*-value	HR (95% CI)	*p*-value
Age (years)				
≤65	1		1	
>65	1.495 (1.311–1.705)	<0.001	1.348 (1.176–1.546)	<0.001
Sex				
Male	1			
Female	1.162 (0.980–1.378)	0.084		
Race				
White	1		1	
Black	2.057 (1.645–2.572)	<0.001	1.333 (1.044–1.701)	0.021
Others	1.251 (0.940–1.666)	0.124	0.941 (0.694–1.276)	0.695
Tumor length (cm)				
≤2	1		1	
>2	2.038 (1.787–2.324)	<0.001	1.465 (1.264–1.697)	<0.001
Histology				
Adenocarcinoma	1		1	
Squamous	1.908 (1.651–2.205)	<0.001	1.212 (0.989–1.486)	0.064
Grade				
G1/G2	1		1	
G3/G4	1.485 (1.300–1.697)	<0.001	1.194 (1.037–1.375)	0.014
Tumor location				
Upper 1/3	1		1	
Middle 1/3	0.794 (0.557–1.132)	0.203	0.852 (0.595–1.219)	0.381
Lower 1/3	0.572 (0.409–0.800)	0.001	0.860 (0.598–1.235)	0.413
Overlapping	1.222 (0.765–1.953)	0.402	1.165 (0.717–1.894)	0.537
T1 subtype				
T1a	1		1	
T1b	0.875 (0.768–0.996)	0.044	1.018 (0.885–1.170)	0.805
Lymph node status				
N0	1		1	
N^+^	2.109 (1.824–2.438)	<0.001	1.242 (1.043–1.478)	0.015
M status				
M0	1		1	
M1	5.380 (4.427–6.540)	<0.001	2.537 (2.032–3.168)	<0.001
Treatment				
None or LTD*	1		1	
S alone	0.516 (0.426–0.626)	<0.001	0.481 (0.392–0.589)	<0.001
CT/RT/CRT	2.716 (2.235–3.301)	<0.001	1.405 (1.115–1.770)	0.004
S plus CT/RT/CRT	0.777 (0.613–0.984)	0.036	0.581 (0.445–0.757)	<0.001

**Table 4:** Cox regression analysis of the risk factors for CSS in patients with survival data.

	Univariate analysis		Multivariate analysis	
	HR (95% CI)	*p*-value	HR (95% CI)	*p*-value
Age (years)				
≤65	1		1	
>65	1.359 (1.152–1.603)	<0.001	1.206 (1.013–1.435)	0.035
Sex				
Male	1		1	
Female	1.293 (1.051–1.591)	0.015	1.060 (0.847–1.327)	0.609
Race				
White	1		1	
Black	2.350 (1.805–3.058)	<0.001	1.286 (0.961–1.720)	0.091
Others	1.377 (0.973–1.951)	0.071	0.943 (0.649–1.370)	0.758
Tumor length (cm)				
≤2	1		1	
>2	2.784 (2.345–3.305)	<0.001	1.800 (1.485–2.183)	<0.001
Histology				
Adenocarcinoma	1		1	
Squamous	2.359 (1.982–2.808)	<0.001	1.408 (1.097–1.807)	0.007
Grade				
G1/G2	1		1	
G3/G4	1.794 (1.520–2.117)	<0.001	1.352 (1.135–1.610)	0.001
Tumor location				
Upper 1/3	1		1	
Middle 1/3	0.836 (0.543–1.289)	0.419	0.887 (0.573–1.374)	0.592
Lower 1/3	0.515 (0.341–0.778)	0.002	0.854 (0.546–1.335)	0.488
Overlapping	1.686 (0.989–2.873)	0.055	1.512 (0.864–2.644)	0.148
T1 subtype				
T1a	1		1	
T1b	0.826 (0.701–0.974)	0.023	0.961 (0.805–1.146)	0.657
Lymph node status				
N0	1		1	
N^+^	2.553 (2.143–3.042)	<0.001	1.246 (1.009–1.538)	0.041
M status				
M0	1		1	
M1	6.861 (5.513–8.539)	<0.001	2.889 (2.240–3.727)	<0.001
Treatment				
None or LTD	1		1	
S alone	0.557 (0.429–0.724)	<0.001	0.496 (0.377–0.653)	<0.001
CT/RT/CRT	3.470 (2.698–4.463)	<0.001	1.403 (1.046–1.880)	0.024
S plus CT/RT/CRT	1.031 (0.763–1.393)	0.842	0.678 (0.484–0.948)	0.023

### Risk stratification of the nomogram

**Figure 7:**
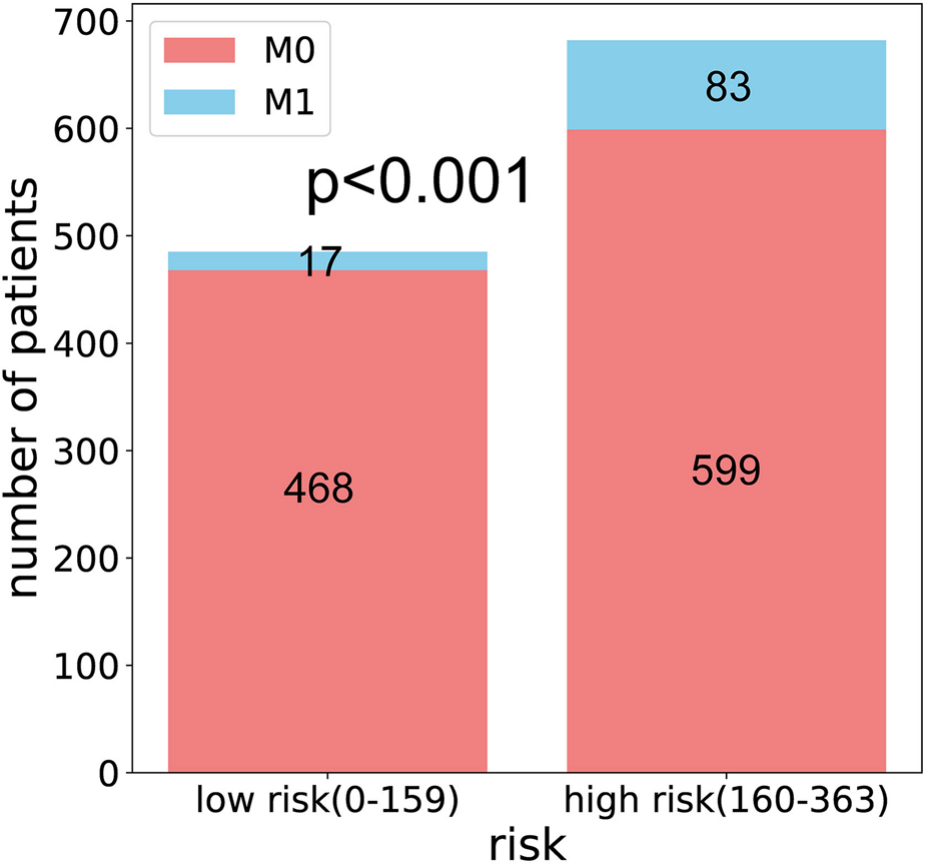
Column chart for risk stratification of the nomogram.

We calculated the median of the nomogram score and divided the cohort into two subgroups (low-risk: 0–159; high-risk: 160–363). In the low-risk group, 468 patients had M0 and 17 patients had M1. In the high-risk group, there were 599 and 89 M0 and M1 patients, respectively. The probability of DM among these subgroups was significantly different (*p *< 0.001) (Figure 7).

### Analysis of different DM sites in 64 patients

**Figure 8:**
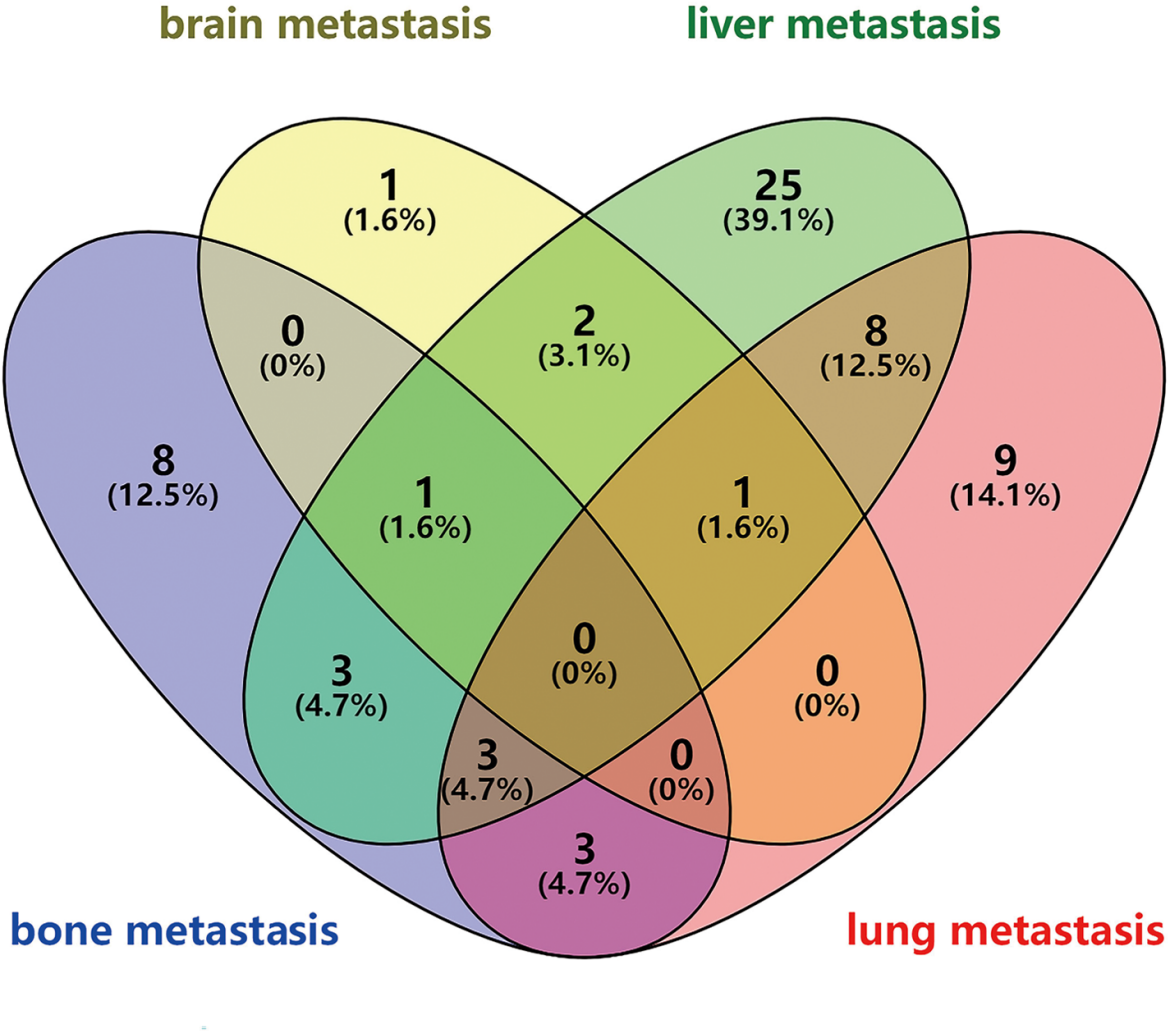
Venn diagram of different organ metastasis.

We performed further analysis of 64 patients with complete DM site data. The Venn diagram indicated the four different proportions of organ metastasis (Figure 8). The most common site of DM was the liver (*n* = 43, 67.2%), followed by the lung (*n* = 24, 37.5%), bone (*n* = 18, 28.1%) and brain (*n* = 5, 7.8%). Furthermore, multiple organ metastasis occurred in 21 patients, including 16 patients with metastases in two organs and 5 patients with metastases in three organs.

## Discussion

Because of its high degree of malignancy, the overall five-year survival of EC ranges from only 15%–25% and is related to the failure to diagnose the disease early in most cases (10). DM is an important factor affecting the treatment and prognosis of patients with EC. Many patients have DM at diagnosis (11), but DM rarely occurs in the T1 stage. We did not find any data on DM in T1 EC in previous studies. In our study, the metastasis rate was approximately 8.6%. Patients with T1 EC usually do not have any symptoms, and most cases are discovered accidentally during gastroscopy. For early T stage patients, clinicians may ignore the possibility of DM, resulting in meaningless surgical resection, insufficient follow-up and postoperative comprehensive treatment. ^18^F-fluorodeoxyglucose PET (FDG-PET) is more sensitive than CT in the detection of DM disease and may improve the detection of DM that may occur on CT (12). However, due to its high price, it is not a suitable examination for all patients, especially for patients with stage T1 disease. Therefore, it is necessary to determine the population with high-risk metastatic characteristics. In this population-based study, we constructed and validated a novel nomogram based on clinicopathological characteristics for the first time to predict DM in patients with stage T1 EC.

Based on the results of statistical analysis, we found that the following five factors were closely related to the DM of T1 EC. According to the contribution to the outcome event, tumor location, lymph node status, tumor length, T1 subtype, and grade were ranked from high to low. Regarding the evaluation of the nomogram, both internal validation and external validation showed good performance. The AUC values in the training cohort and validation cohort were both over 0.8, proving that the model has a good degree of discrimination. The H-L test combined with the calibration plot in the training cohort and validation cohort both proved that the model had good calibration. Furthermore, we divided patients into two risk groups based on nomogram scores. It can better help clinicians identify which patients need to be treated more cautiously. Furthermore, this model may allow clinicians to closely follow high-risk patients so that appropriate adjuvant treatment can be provided in time.

Among all predictors, tumor location made the largest contribution. We found that compared with tumors located in the upper 1/3 of the esophagus, patients with overlapping lesions (OR = 7.948, 95% CI: 1.372–46.060, *p *= 0.021) and tumors located in the lower 1/3 of the esophagus (OR = 4.431, 95% CI: 0.896–21.910, *p *= 0.068) were more likely to develop DM. A previous study obtained the same result, but they did not limit the study population to stage T1 (13). According to the ICD-O-3 codes, overlapping lesions were defined as tumors that overlapped the boundaries of two or more subcategories and whose point of origin could not be determined. We could not find relevant evidence regarding to why tumors that overlapped and those in the lower 1/3 of the esophagus have a higher tendency toward DM. We speculate that this may be related to the organ metastasis pattern of EC. Our data suggested that the liver is the most common site for DM of EC, followed by the lung, which is consistent with previous research (14, 15). In the present study, tumors located in the lower 1/3 accounted for the vast majority (74.3%). The blood in the distal segments of the esophagus mainly drains into the left gastric vein and then drains into the portal vein. This may explain the phenomenon we observed.

Lymph node status was also a key factor. Many studies have found that lymph node-related indicators were associated with DM and survival. A study by Sakanaka et al. (16) was designed to verify that a larger lymph node size was associated with a high risk of DM and poor OS in EC patients after definitive chemoradiotherapy. Huang et al. (17) retrospective 167 EC patients with regional lymph node metastasis and found that a positive lymph node ratio (PLNR) greater than 0.15 had an increased risk of postoperative DM and decreased OS. In our study, we found that T1 EC patients with N^+^ had a 4.6-fold increase in the risk of DM compared with patients with N0. Similarly, lymph node status was also an independent prognostic factor in T1 EC patients (OS: N0 vs. N^+^, HR = 1.44, *p *< 0.001; CSS: N0 vs. N^+^, HR = 1.46, *p* < 0.001).

Many previous studies (18–20) proved that longer tumor lengths were associated with poor survival in EC patients. In the present study, we also found that this factor was an independent prognostic factor for OS and CSS in T1 EC patients. In addition, patients with tumor lengths greater than 2 cm were more likely to develop DM. The larger the area covered by the tumor is, the greater the chance of tumor cells entering the blood. Poor histologic grade usually indicates a stronger invasive and metastatic ability. Compared with G1/G2 patients, patients with G3/G4 had a higher risk of DM. Similar results can be found in other solid tumor studies (21–23). Moreover, what surprised us was that patients with T1a were more prone to DM than those with T1b. We speculated that this may be related to two reasons. On the one hand, the deletion of some patients with the T1 subtype was unknown at the time of enrollment. On the other hand, clinical staging was used for many patients with DM. In the SEER database, it is not clear whether the AJCC stage for each patient is pathological or clinical. In our opinion, if they were treated with surgery, the stage is pathological; conversely, it is clinical. In our training cohort, 69 (69%) patients with M1 were stage T1a. However, most patients did not receive surgical treatment, which means that the staging of M1 patients is almost always clinical. Therefore, it is difficult to accurately distinguish T1a and T1b by imaging. This may lead to some T1b tumors being misidentified as T1a. In summary, it may result in a bias in the results. However, the T1 subtype did not contribute much to the model, with little impact on the predictive results.

To more easily identify high-risk patients with DM, we divided the patients into two groups according to the median of the nomogram score. Among them, patients with scores of 160–363 were regarded as the high-risk group for DM. For these patients, PET-CT scans should be considered before surgery if they can afford them.

In this population-based study, we rigorously screened 1,663 eligible patients from real-world data. After appropriate statistical analysis, we obtained these convincing results. However, some limitations exist in our study. First, given the retrospective nature of the study, some bias is inevitable. Second, lymphovascular (LVI) and perineural (PNI) invasion were proven to be associated with an increased incidence of DM (24). The SEER database lacks data on these two variables. The efficacy of the nomogram may be further improved if LVI and PNI are included. Third, the model was not verified by an external cohort. In the future, multicenter data should collected, and other factors can be added to improve the model.

## Conclusion

In conclusion, we constructed and validated a novel nomogram containing five clinicopathological factors to accurately predict DM in T1 EC. The model showed high discrimination, calibration and clinical application value. This tool can help clinicians better identify high-risk DM patients and guide clinical decisions.

## Data Availability

The datasets presented in this study can be found in online repositories. The names of the repository/repositories and accession number(s) can be found below: http://seer.cancer.gov/.
