# Supplementary material for: Risk factors for distant metastasis and prognosis in stage T1 esophageal cancer: A population-based study
Source: Front Surg. 2023 Jan 6;9:988460. doi: 10.3389/fsurg.2022.988460 (PMC9852716; doi:10.3389/fsurg.2022.988460)
Supplement: Supplementary file 1 [file Table1.docx]

Table S1: Clinicopathologic characteristics of T1 EC patients

| Variables | Whole cohort  (n=1663) | Training cohort  (n=1167) | Validation cohort  (n=496) | *p value* |
| --- | --- | --- | --- | --- |
| **Age (years)** |  |  |  | 0.301 |
| ≤65 | 836 (50.3) | 577 (49.4) | 259 (52.2) |  |
| >65 | 827 (49.7) | 590 (50.6) | 237 (47.8) |  |
| **Sex** |  |  |  | 0.215 |
| Male | 1386 (83.3) | 964 (82.6) | 422 (85.1) |  |
| Female | 277 (16.7) | 203 (17.4) | 74 (14.9) |  |
| **Race** |  |  |  | 0.148 |
| White | 1466 (88.2) | 1033 (88.5) | 433 (87.3) |  |
| Black | 115 (6.9) | 84 (7.2) | 31 (6.3) |  |
| Others | 82 (4.9) | 50 (4.3) | 32 (6.4) |  |
| **Tumor length (cm)** |  |  |  | 0.247 |
| ≤2 | 896 (53.9) | 618 (53.0) | 278 (56.0) |  |
| >2 | 767 (46.1) | 549 (47.0) | 218 (44.0) |  |
| **Histology** |  |  |  | 0.038 |
| Adenocarcinoma | 1304 (78.4) | 931 (79.8) | 373 (75.2) |  |
| Squamous | 359 (21.6) | 236 (20.2) | 123 (24.8) |  |
| **Grade** |  |  |  | 0.441 |
| G1/G2 | 1097 (66.0) | 763 (65.4) | 334 (67.3) |  |
| G3/G4 | 566 (34.0) | 404 (34.6) | 162 (32.7) |  |
| **Tumor location** |  |  |  | 0.232 |
| Upper 1/3 | 54 (3.2) | 34 (2.9) | 20 (4.0) |  |
| Middle 1/3 | 326 (19.6) | 224 (19.2) | 102 (20.6) |  |
| Lower 1/3 | 1236 (74.3) | 871 (74.6) | 365 (73.6) |  |
| Overlapping | 47 (2.9) | 38 (3.3) | 9 (1.8) |  |
| **T1 subtype** |  |  |  | 0.534 |
| T1a | 859 (51.7) | 597 (51.2) | 262 (52.8) |  |
| T1b | 804 (48.3) | 570 (48.8) | 234 (47.2) |  |
| **Lymph node status** |  |  |  | 0.758 |
| N0 | 1303 (78.4) | 912 (78.1) | 391 (78.8) |  |
| N^+^ | 360 (21.6) | 255 (21.9) | 105 (21.2) |  |
| **M status** |  |  |  | 0.947 |
| M0 | 1520 (91.4) | 1067 (91.4) | 453 (91.3) |  |
| M1 | 143 (8.6) | 100 (8.6) | 43 (8.7) |  |

Table S2: Baseline characteristics between patients with T1N0M1 and T1N^+^M1 (n=143)

| Variables | T1 N0 M1  (n=58) | T1 N^+^ M1  (n=85) | *p value* |
| --- | --- | --- | --- |
| **Age (years)** |  |  | 0.474 |
| ≤65 | 32 (38.1) | 26 (44.1) |  |
| >65 | 52 (61.9) | 33 (55.9) |  |
| **Sex** |  |  | 0.232 |
| Male | 47 (81.0) | 75 (88.2) |  |
| Female | 11 (19.0) | 10 (11.8) |  |
| **Race** |  |  | 0.369 |
| White | 46 (79.3) | 70 (82.4) |  |
| Black | 10 (17.2) | 9 (10.5) |  |
| Others | 2 (3.5) | 6 (7.1) |  |
| **Tumor length (cm)** |  |  | 0.054 |
| ≤2 | 13 (22.4) | 9 (10.6) |  |
| >2 | 45 (77.6) | 76 (89.4) |  |
| **Histology** |  |  | 0.813 |
| Adenocarcinoma | 42 (72.4) | 60 (70.6) |  |
| Squamous | 16 (27.6) | 25 (29.4) |  |
| **Grade** |  |  | 0.031 |
| G1/G2 | 31 (53.4) | 30 (35.3) |  |
| G3/G4 | 27 (46.6) | 55 (64.7) |  |
| **Tumor location** |  |  | 0.010 |
| Upper 1/3 | 0 (0.00) | 3 (3.5) |  |
| Middle 1/3 | 10 (17.2) | 12 (14.2) |  |
| Lower 1/3 | 48 (82.8) | 58 (68.2) |  |
| Overlapping | 0 (0.00) | 12 (14.1) |  |
| **T1 subtype** |  |  | 0.256 |
| T1a | 44 (75.9) | 57 (67.1) |  |
| T1b | 14 (24.1) | 28 (32.9) |  |

Table S3: Nomogram score of independent factors for DM

| Variables | Score |
| --- | --- |
| **Tumor length (cm)** |  |
| ≤2 | 0 |
| >2 | 78 |
| **Grade** |  |
| G1/G2 | 0 |
| G3/G4 | 38 |
| **Tumor location** |  |
| Upper 1/3 | 0 |
| Middle 1/3 | 18 |
| Lower 1/3 | 62 |
| Overlapping | 100 |
| **T1 subtype** |  |
| T1a | 60 |
| T1b | 0 |
| **Lymph node status** |  |
| N0 | 0 |
| N^+^ | 87 |
